# Regional differences in WT-1 and Tcf21 expression during ventricular development: implications for myocardial compaction

**DOI:** 10.1371/journal.pone.0136025

**Published:** 2015-09-21

**Authors:** Rebecca Vicente-Steijn, Roderick W. C. Scherptong, Boudewijn P. T. Kruithof, Sjoerd N. Duim, Marie Jose T. H. Goumans, Lambertus J. Wisse, Bin Zhou, William T. Pu, Robert E. Poelmann, Martin J. Schalij, Michelle D. Tallquist, Adriana C. Gittenberger-de Groot, Monique RM Jongbloed

**Affiliations:** 1 Department of Cardiology, Leiden University Medical Center, Leiden, The Netherlands; 2 Department of Anatomy and Embryology, Leiden University Medical Center, Leiden, The Netherlands; 3 Department of Molecular Cell Biology, Leiden University Medical Center, Leiden, The Netherlands; 4 Center for Cardiovascular Research, John A. Burns School of Medicine, University of Hawaii, Honolulu, Hawaii, United States of America; 5 Key Laboratory of Nutrition and Metabolism, Institute for Nutritional Sciences, Shanghai Institutes for Biological Sciences, Graduate School of the Chinese Academy of Sciences, Chinese Academy of Sciences, Shanghai, China; 6 Harvard Stem Cell Institute, Harvard University, Cambridge, and Department of Cardiology, Boston Children's Hospital, Boston, Massachusetts, United States of America; San Raffaele Pisana, ITALY

## Abstract

**Background:**

Morphological and functional differences of the right and left ventricle are apparent in the adult human heart. A differential contribution of cardiac fibroblasts and smooth muscle cells (populations of epicardium-derived cells) to each ventricle may account for part of the morphological-functional disparity. Here we studied the relation between epicardial derivatives and the development of compact ventricular myocardium.

**Results:**

Wildtype and Wt1^CreERT2/+^ reporter mice were used to study WT-1 expressing cells, and Tcf21^lacZ/+^ reporter mice and PDGFRα^-/-^;Tcf21^LacZ/+^ mice to study the formation of the cardiac fibroblast population. After covering the heart, intramyocardial WT-1+ cells were first observed at the inner curvature, the right ventricular postero-lateral wall and left ventricular apical wall. Later, WT-1+ cells were present in the walls of both ventricles, but significantly more pronounced in the left ventricle. Tcf21-^LacZ^ + cells followed the same distribution pattern as WT-1+ cells but at later stages, indicating a timing difference between these cell populations. Within the right ventricle, WT-1+ and Tcf21-lacZ+ cell distribution was more pronounced in the posterior inlet part. A gradual increase in myocardial wall thickness was observed early in the left ventricle and at later stages in the right ventricle. PDGFRα^-/-^;Tcf21^LacZ/+^ mice showed deficient epicardium, diminished number of Tcf21-^LacZ^ + cells and reduced ventricular compaction.

**Conclusions:**

During normal heart development, spatio-temporal differences in contribution of WT-1 and Tcf21-^LacZ^ + cells to right versus left ventricular myocardium occur parallel to myocardial thickening. These findings may relate to lateralized differences in ventricular (patho)morphology in humans.

## Introduction

Right ventricular (RV) function is an important determinant of survival in cardiovascular diseases [1]. Therapies aimed at long-term improvement of RV function are scarce [2], and therapies beneficial in left ventricular (LV) disease are in general less effective for the dysfunctional RV [3,4]. Therefore, development of dedicated therapies might be of interest for the treatment of specific RV diseases [5]. Proper understanding of the morphological and molecular differences between the LV and RV is mandatory to develop therapeutic options directed at RV dysfunction.

Early in development the heart consists of a primary heart tube [6], and through migratory processes cells are added from the second heart field (SHF) to the arterial and venous poles of the heart [7–9]. Whereas the primary heart tube contains the majority of cells of the LV, the SHF provides most components of the RV [8,10]. This different origin (primary heart tube versus SHF) and timing (early LV versus later RV) may reflect observed differences between the adult LV and RV. The normal adult LV has a smooth interventricular septum and a thicker compact myocardial layer as compared to the adult RV. The normal adult RV is characterized by the presence of a trabecula septomarginalis and a moderator band and trabeculations are coarser [11]. Many morphologists contemplate a so-called tripartite architecture of the ventricles, divided in an inlet, an apical, and an outlet part [11], being relevant in specific congenital heart diseases involving hypoplasia of one of those elements [12].

The proepicardial organ (PEO), is a temporary cluster of cells located caudal of the developing heart that will give rise to the epicardial cell layer. Epicardial cells covering the distal vascular part of the outflow tract (OFT) originate from the arterial pole of the heart [13]. After spreading over the heart, epicardial cells undergo epithelial-to-mesenchymal transition (EMT), form a subepicardial layer and migrate subsequently into the ventricular wall as epicardium derived cells (EPDCs) [14]. EPDCs contribute to coronary vessel formation, differentiation of the Purkinje network, ventricular septation [15] and differentiate into interstitial fibroblasts [16–18]. The latter cell-population induces normal LV growth [19]. Knock-out of epicardial-associated genes showed abnormal epicardium and abnormal formation and compaction of the ventricular myocardium[20–22].

Several markers exist to identify the epicardium and its derived cells. Wilms tumor 1(WT-1), one such marker, has a high specificity for epicardial cells and early EPDCs [23]. WT-1+ cells have been shown to contribute mostly to interstitial fibroblasts and smooth muscle cells [24]. Expression of WT-1 is found later in cells of the endothelial lineage [25–27]. Recently, the role of the basic helix-loop-helix transcription factor Tcf21 in lineage specification of epicardial cells has been described. Tcf21 is expressed early in the PEO and later in the epicardium and EPDCs. Tcf21+ cells are initially able to contribute to both (smooth muscle and fibroblast) lineages, however at EMT stages the majority of Tcf21 expressing cells become restricted to the fibroblast lineage [18]. Additional factors control cardiac fibroblast development, such as PDGFRα, which is required for migration and EMT of EPDCs [17] and is crucial for their differentiation [28]. Knock-out of PDGFRα is associated with thin uncompacted myocardium [21]. To date, there are no reports comparing the differences in distribution of WT-1 and Tcf21 expression in right and left ventricular myocardium during development.

The aim of the current study is to analyse the differences in myocardial architecture specifically between the RV and the LV in relation to epicardial formation and distribution of WT-1+ cells and Tcf21+ cells. Therefore, it was assessed whether timing and distribution of WT-1 and Tcf21^LacZ^ expressing cells entering the ventricle varies for RV and LV and within the RV itself, as anatomical evidence suggests that the RV can be divided into a posterior (inlet) and anterior (outlet) portion.

## Results

### Specific spatiotemporal epicardial covering of the early embryonic heart

**Fig 1:**
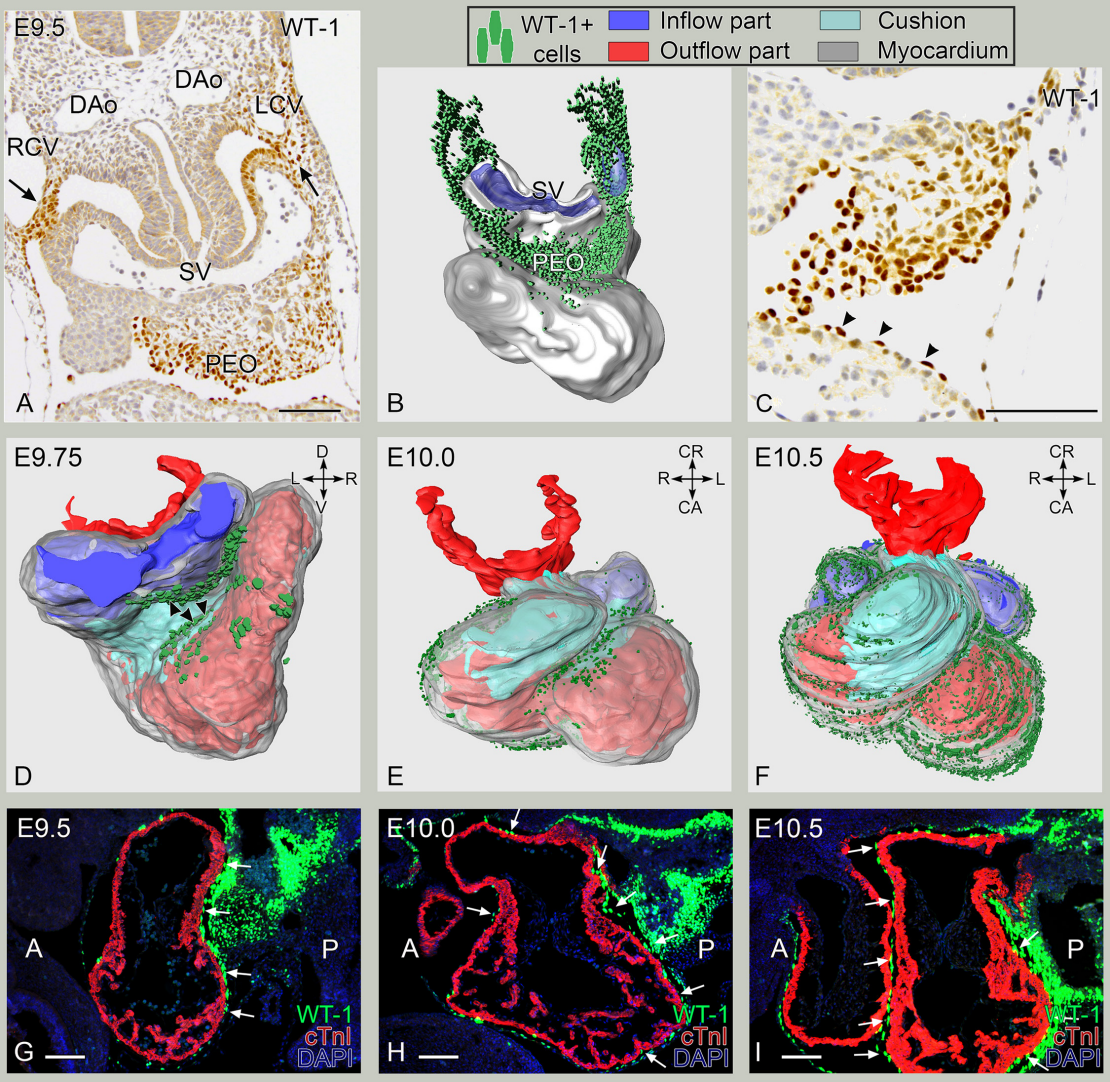
E9.5-E10.5. The proepicardial organ and the first covering of the embryonic heart. A: Transverse section. Caudal of entrance of the right (RCV) and left (LCV) cardinal veins in the sinus venosus (SV). WT-1 expression in tissues surrounding LCV and RCV (arrows in A) is continuous with the proepicardial organ (PEO). B: 3D-reconstruction of embryonic day (E) 9.5 mouse heart (myocardium depicted in grey, (caudal view) in which the WT-1+ nuclei have been superimposed in green. C: Transverse section. PEO cells express WT-1 (A,C) and the first epicardial cells(arrowheads) attach to the surface of the ventricle. D-F: 3D reconstructions of E9.75, E10.0 and E10.5 respectively depicting the first steps of epicardial covering (epicardial WT-1+ cell population in green) going from dorsal to ventral and from caudal to cranial. D shows a caudal view of the heart in which the first WT-1+ nuclei are observed in the posterior region of the ventricles and lower region of the atria. E. E10.0 reconstruction showing the epicardial covering starting to reach the anterior side of the heart. F. E10.5 reconstruction shows that the covering of the ventricles is finalized except for the outflow tract region of the heart. G-H: Sagital sections. Early epicardial covering of the heart (white arrows) occurs in a posterior (P) to anterior (A) manner as shown by fluorescence double stainings of WT-1 (green) and cTnI (red) at E9.5 (G), E10.0 (H) and E10.5 (I). DAo: dorsal aorta. Bars: 100 μm

The PEO is an embryonic structure from which the epicardial cells migrate to cover the developing heart, that is initially devoid of epicardium. The PEO could be recognized at embryonic day (E)9.5 as a slightly left-sided structure protruding into the coelomic cavity and expressing WT-1 (Fig 1A). Next to expression in the PEO, WT-1 expression was observed in the mesenchyme surrounding the left and right cardinal veins (Fig 1A, arrows). A 3D reconstruction of sections was made (Fig 1B), and showed that these WT-1+ cells formed a semicircle around the left and right cardinal veins and the proximal part of the sinus venosus. The first WT-1+ cells covering the myocardium became visible at the inner curvature, covering the infero-posterior part of the atria and ventricles (Fig 1B–1D (arrowheads)). The primitive LV was larger than the primitive RV and trabeculations were observed in both. The outer compact layer of myocardium consisted of 1–2 cell layers. 3D reconstructions of stages E9.75, E 10 and E10.5 (Fig 1D–1F) were made to study regional differences in this gradual covering of the heart by WT-1+ cells (S1 File). By E10.0, the posterior side of both atria and ventricles were covered by WT-1+ cells, whereas the anterior part of the ventricles was largely uncovered with exception of the area in proximity to the inner curvature (Fig 1E, also see S1 File, button E10.0). By E10.5, the majority of the LV was covered by WT-1+ cells as wells as the posterior part of the RV. Covering of the anterior part of the RV appeared less dense as compared to covering of the posterior part of the RV and the LV (compare RV and LV views in the S1 File, button E10.5). As in previous stages, the developing outflow tract (OFT) was still largely depleted of WT-1+ epicardial cells (Fig 1F, S1 File, button E10.5). Thus, the covering of the heart occurred between E9.5 and E10.5, starting from the caudally located PEO, in a dorsal to ventral fashion, as was confirmed by sagittal sections of the developing heart (Fig 1G–1I).

### The outflow tract (OFT) is covered the latest in an asymmetric fashion

**Fig 2:**
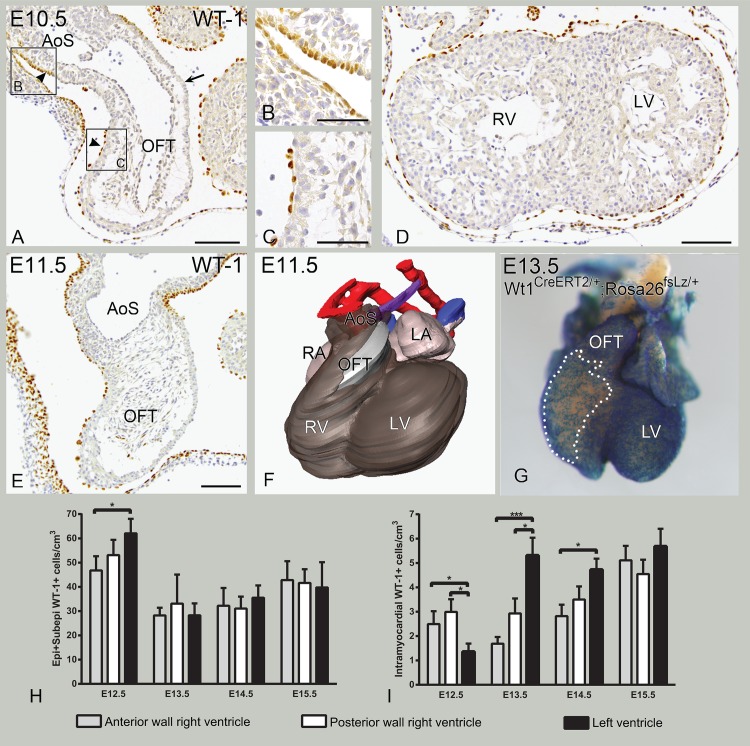
E10.5–12.5. Epicardial covering of the heart. A-D: E10.5, transverse sections. A. Outflow tract (OFT) covered with WT-1+ cells on the putative aorta (right side), but not on the putative pulmonary trunk (left side, arrow). Arterial pole epicardium is cuboidal (arrowhead in upper box in A, enlarged in B). Epicardial cells covering the proximal right part of OFT are squamous (arrowhead in lower box in A, enlarged in C). D. Towards the apex both right ventricle (RV) and left ventricle (LV) have an epicardial covering. The LV is fully covered by epicardium. E-G: E. Transverse section. At E11.5 the left side of the OFT is uncovered. F. E11.5 3D-reconstruction of myocardium (grey for RV and OFT, pink for atria) and epicardium (brown). The lumen of the aorta is depicted in red and the lumen of the cardinal veins in blue. The putative pulmonary trunk (PT, purple) is the last to become covered by epicardium. G: E13.5, Rosa26^fsLz/+^, whole mount. PEO-derived epicardium is fully developed; there is a lower density of epicardial cells at the anterior side of the RV than LV. H-I: Ventricular quantification of WT-1+ cells per ventricular region is shown. H shows the epicardial and subepicardial portion of WT-1+ cells per cm^3^ for the different regions of the ventricles in different stages of development. I shows the intramyocardial WT-1+ cell population per cm^3^. *p<0.05; ***p<0.001; ****p<0.0001. AoS: aortic sac; LA: left atrium, RA: right atrium. Bars: panels B,C,F,G:50μm, panels A,D,E,H,I:100 μm

As previously shown, the epicardial covering of the heart follows a specific order: from dorsal to ventral and from caudal to cranial. It is therefore logical that the OFT area will be the last structure to get fully covered by an epicardial layer. However, it is interesting to note that this happens in an asymmetric fashion. WT-1 expression was more prominent on the right, putative aortic, side (Fig 2A–2C), as compared to the left side, the putative pulmonary trunk (arrow in Fig 2A). Furthermore, the arterial epicardium [13,16] that covered the distal segment of the OFT was cuboid in shape (Fig 2A and 2B), as compared to the PEO-derived epicardium covering part of the left proximal segment of the OFT (Fig 2A and 2C). Later in development (E11.5 to E13.5), the PEO-derived epicardium extended further towards the distal segment of the OFT, connecting on the aortic side to the arterial pole-derived epicardium to form a continuous epithelium, whereas the left (putative pulmonary trunk) side was still largely uncovered (Fig 2E–2G).

### Epicardial covering and intramyocardial patterning of WT-1+ cells differs between ventricles

**Fig 3:**
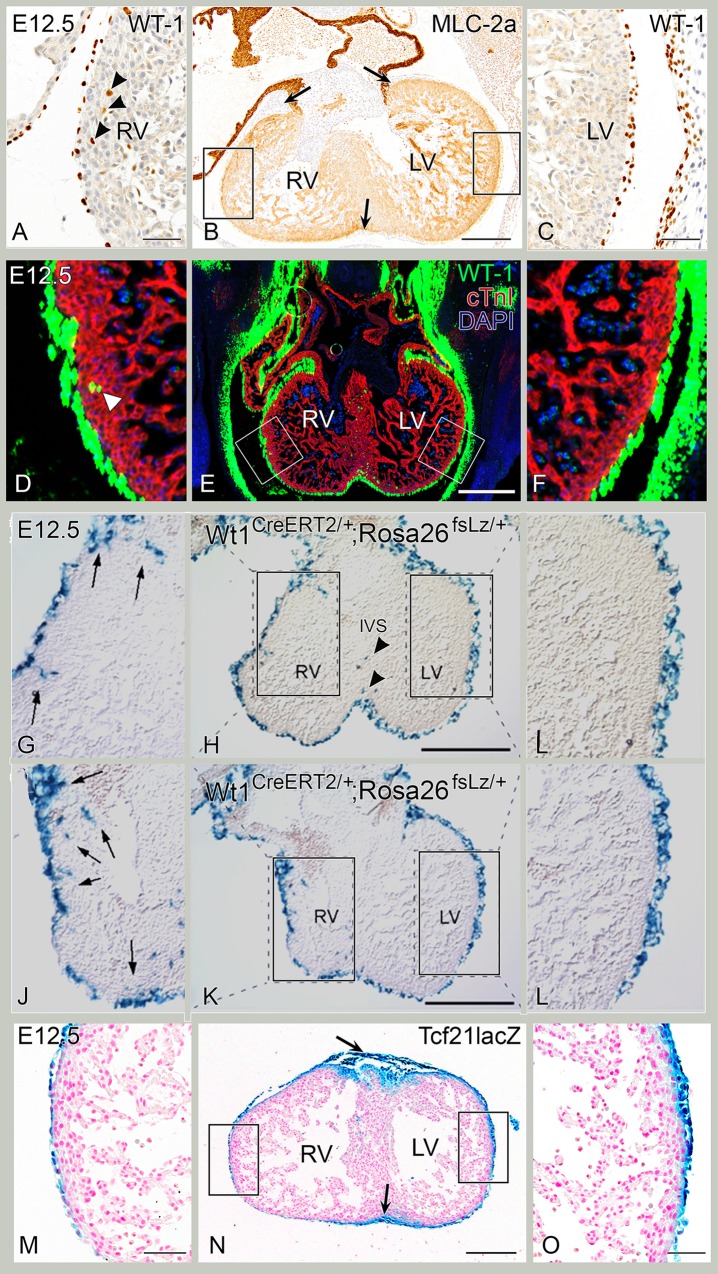
E12.5. Early intramyocardial WT-1 positive cells. A-C. Control, transverse sections. B. E12.5 MLC-2a staining of right ventricle (RV) and left ventricle (LV) with complete epicardial covering and a visible subepicardial space in the LV and in the atrioventricular and interventricular sulcus (arrows). A, C: Enlargements of adjacent WT-1 stained sections of boxes in B, showing RV (A) and LV (C). The subepicardial layer is slightly broader on the LV side. WT-1+ cells are found in RV lateral wall (B, arrowheads) but not in the LV lateral wall (C). D-F: Fluorescent double stainings of WT-1 (green) and cTnI (red) at E12.5 confirm the first WT-1+ cells in the RV lateral wall, in the apical LV wall and the interventricular septum. G-L: WT1^CreERT2/+^, E12.5. There is a subepicardial layer in both ventricles with WT-1+ cells in the RV lateral wall (arrows in G and J), but not at the LV lateral wall (I and L). Some WT-1+ cells present at the base of both ventricles, and in the interventricular septum (IVS) (arrowheads in H). M-O: Tcf21^lacZ/+^ mouse sections stained for LacZ in blue. N. LacZ staining of RV and LV comparable to B, showing epicardial covering and subepicardial space in the LV and in the atrioventricular and interventricular sulcus (arrows). M,O: Enlargements of boxed areas in E, showing RV (M) and LV (O). As in C, the subepicardial layer is slightly broader compared to the RV. No LacZ + cells where found in the RV lateral wall. Bars: B,E: 200μm, H, K: 500 μm, other bars: 50 μm.

The ventral side of the primitive RV receives epicardial covering at later stages. At early stages of development (E10.5-E12.5), covering of the body of the RV chamber was less densely covered with WT-1+ cells (Figs 1F, 2D and 2H). At E10.5, the epicardium appeared to connect less tight to the LV than the RV and no subepicardial WT-1+ layer was present in either ventricle yet (Fig 2D). At E12.5 the LV showed a higher density of WT-1+ cells when compared to the RV, specifically the anterior portion (Fig 2H). Although the amount of cells per cm^3^ became comparable between ventricles by E13.5 (Fig 2H), the anterior surface of the RV at stage E13.5 showed a patchy covering of LacZ+ cells (WT-1Cre-ERT2, Fig 2G). A subepicardial space had formed mainly at the atrioventricular and interventricular sulcus (arrows in Fig 3B).

During normal heart development, EMT throughout the epicardium covering the ventricles results in formation of a subepicardial layer containing EPDCs. At E12.5, intramyocardial WT-1+ cells were seen in the inner curvature and the lateral wall of the posterior (inlet part) of the RV, but not yet in the lateral wall of the LV (Fig 3A–3F). Intramyocardial WT-1+ cells were also observed in the apical wall of the LV and in the interventricular septum (Fig 3E **)**. The fraction of epicardial and subepicardial WT-1+ cells and the fraction of intramyocardial WT-1+ cells was quantified at different stages of development (Fig 2H and 2I). At E12.5, quantification revealed a significantly higher cell density of epicardial and subepicardial cells in the LV compared to the anterior region of the RV, but no significant differences between the posterior region of the RV and the LV were observed (Fig 2H). The higher amount in the LV is probably due to the more abundant epicardial and subepicardial layer. After tamoxifen injection at E10.5, LacZ expression at E12.5 from the WT1^CreERT2/+^; Rosa26^fsLacZ/+^, also showed the presence of WT-1 lineage cells in the thin myocardial layer of the RV lateral wall, but none in the LV lateral wall (Fig 3G–3L). This was confirmed by a significant higher intramyocardial WT-1+ cell density in the anterior and posterior region of the RV compared to the LV at E12.5 (Fig 2I). The atria showed epicardial covering but no subepicardial space and no intramyocardial WT-1+ cells. No expression of WT-1 was observed in endothelial cells at this stage (data not shown).

**Fig 4:**
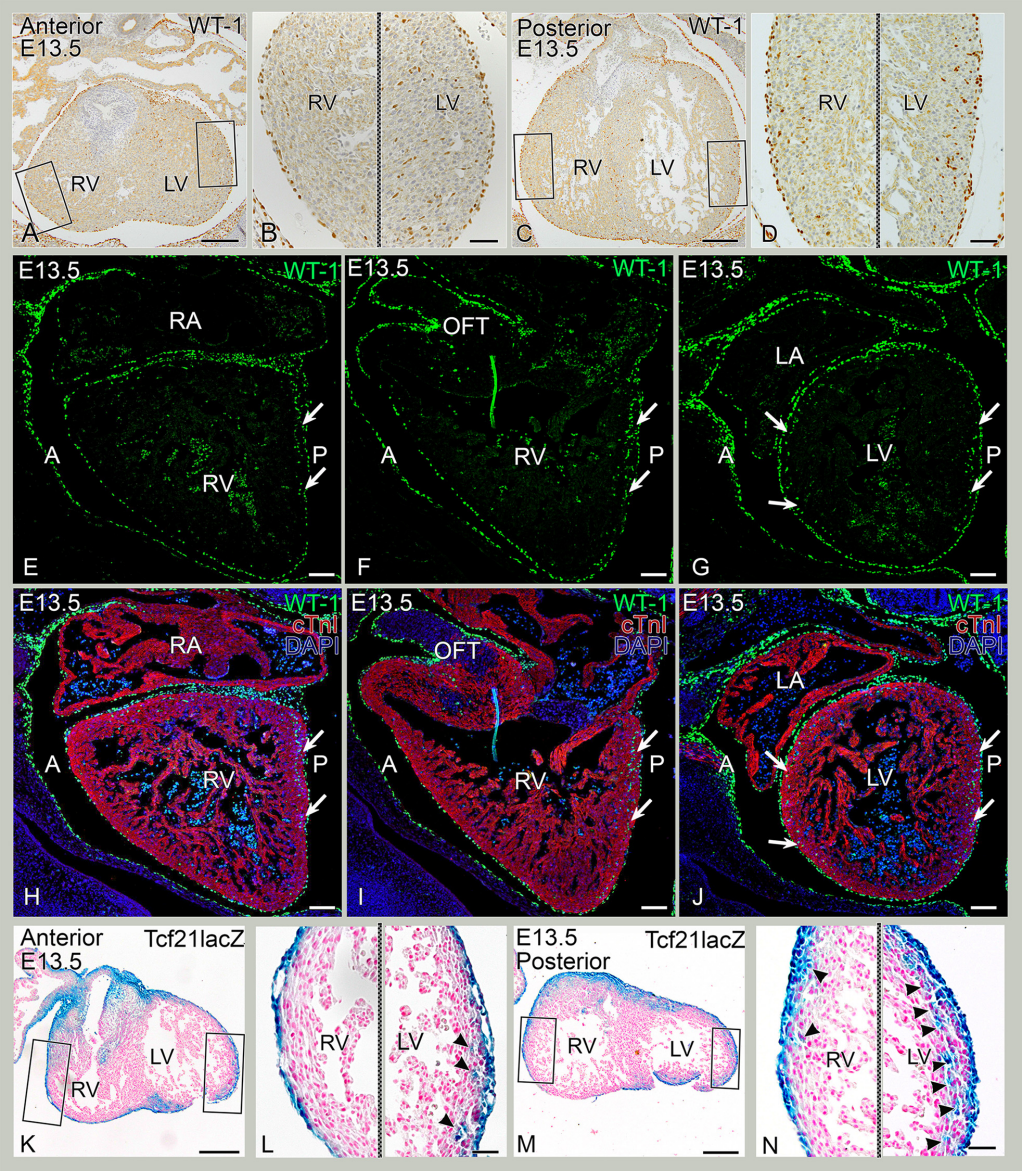
Regional differences in intramyocardial WT-1 expression at E13.5. A-D: Control, transverse sections. A. Anterior section, stained for WT-1. B: Enlargement of the box in A with few WT-1+ cells in the right ventricle (RV) and more WT-1+ cells in the left ventricle (LV). C. Posterior section, stained for WT-1. D: Enlargement of RV in D with more WT-1+ cells than anterior and enlargement of the LV with overall more WT-1+ cells than in the RV (compare with panel B). E-J: fluorescence sequential sagittal sections from RV (E-F, H-I) towards LV (G,J) double stained for WT-1 (green) and cTnI (red). Arrows indicate areas with abundant WT-1+ cells. Anterior (A) and posterior (P) are indicated. K-N: Tcf21^lacZ/+^ mouse sections stained for LacZ in blue. K. Anterior section of the heart. L: Enlargement of RV with no LacZ+ cells and of LV with sporadic WT-1+ cells (arrowheads). M. Posterior section of the heart. N: Enlargement RV with sporadic LacZ+ cells (arrowheads) and of LV with overall more LacZ+ cells than in the RV (arrowheads). Bars: A,C,E,G: 200 μm, B,D,F,H: 50 μm, I-N: 100 μm.

**Fig 5:**
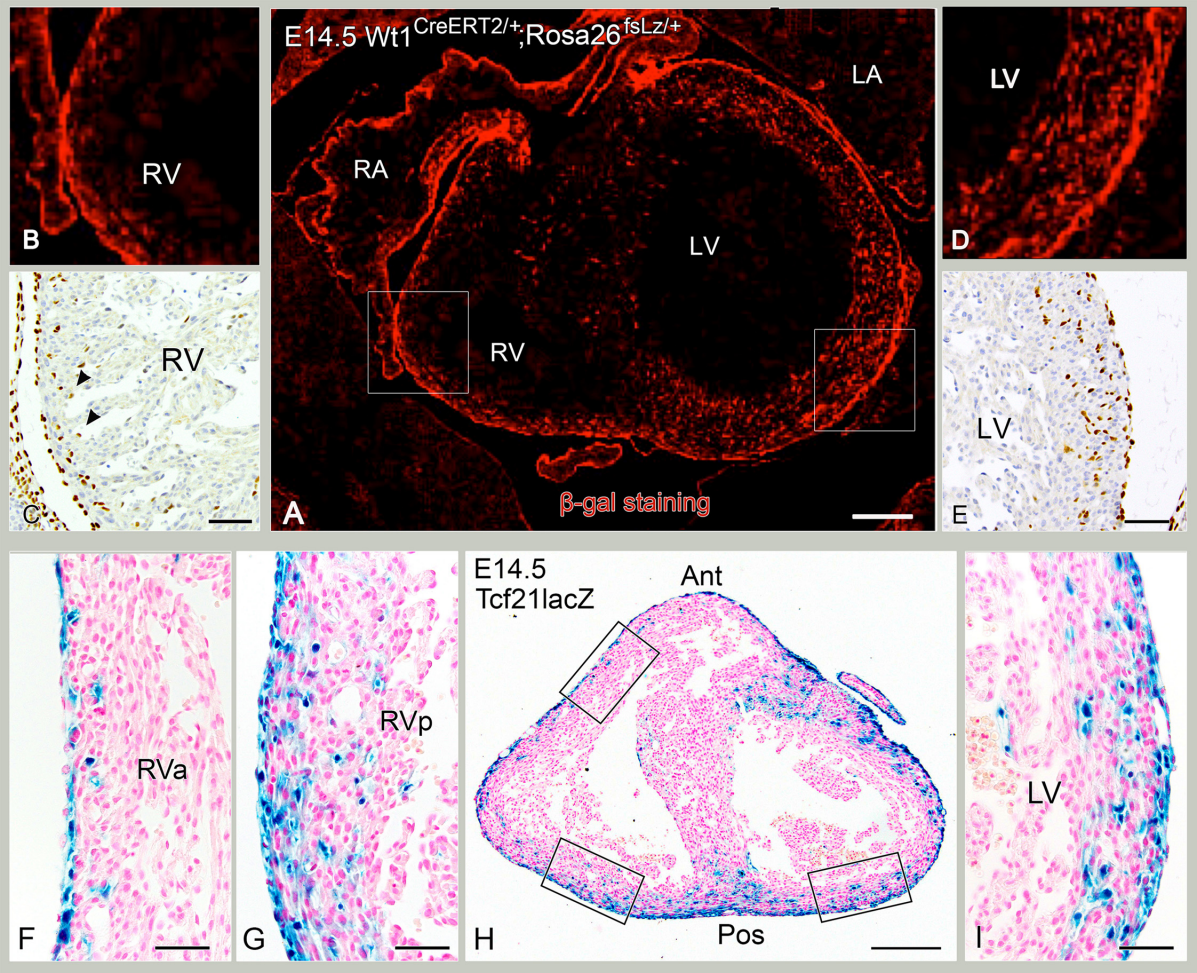
WT1-derived cells and Tcf21^lacZ/+^ show regional differences in intramyocardial distribution of at E14.5. A-E: WT1^CreERT2/+;^ Rosa26^fsLz/+^ mouse section (A,B,D) and control WT-1 staining (C,E) showing differences between RV and LV in WT-1+ cells. Red tissue in A,B,D indicates β-gal staining. A is an overview of RV and LV, B and D are enlargements of RV (B) and LV (D). C and E show WT-1+ cells comparable to B and D. F-I: H: Tcf21^lacZ/+^ mouse section stained for LacZ in blue. Anterior (Ant) and posterior (Pos) are indicated. The upper box at the RV (H) corresponds to the enlargement in F of the anterior RV, the lower box at the RV (H) corresponds to the enlargement (G) of the posterior RV. The box of the LV in H corresponds to enlargement (I). Tcf21^lacZ/+^ demonstrates the same distribution pattern, i.e. more lacZ+ cells in LV than RV (compare G with I) and more lacZ+ cells in posterior part of RV than anterior (compare G with F). Bars: I,N: 200 μm, other bars: 50 μm.

At E13.5, fewer intramyocardial WT-1+ cells were seen in the anterior portion of the RV compared to the LV (Fig 4A and 4B). More posteriorly, progressively more intramyocardial WT-1+ cells in the RV lateral wall were present (Fig 4C and 4D) with less cells apically. Sagital sections of E13.5 wildtype mouse hearts confirmed these observations, showing less WT-1 + cells in the anterior wall of the RV as compared to the posterior wall (Fig 4E–4J). Quantification of the intramyocardial WT-1+ cell population at this stage revealed a significant difference between the LV and the anterior as well as the posterior region of the RV (Fig 2I). At E14.5, this difference in intramyocardial WT-1+ cells was still maintained (Fig 5A–5E) specially for the anterior region of the RV (Fig 2I) while the posterior region of the RV showed more resemblance to the LV, thus a higher cell density was observed in the posterior RV compared to the anterior portion. In conclusion, overall, a higher density of WT-1+ and WT-1 lineage cells were observed in the LV than RV (Figs 4B, 4D and 5A–5E). By E15.5, quantification of WT-1+ cells showed that the differences between the LV and RV had disappeared (Fig 2I, indicating that the amount of cells found in the RV catched up with the LV. The interventricular septum showed relatively more intramyocardial WT-1+ cells throughout development and was not included in the analysis.

### Intramyocardial Tcf21 LacZ+ cell population follows an asymmetric distribution between ventricles

To determine whether the differential WT-1+ cellular contribution to the left and right ventricle is also observed for the fibroblast population, mouse embryos containing the Tcf21^lacZ/+^ allele were studied. Tcf21 is a robust fibroblast lineage marker after EMT [18]. At E12.5, LacZ expression was observed throughout the entire epicardial layer (Fig 3M–3O), as well as in the atrioventricular and interventricular sulcus tissue (Fig 3N). However, at this point, no LacZ expressing cells were identified within the myocardium of either ventricle compared to WT-1+ cells (compare Fig 3G–3L to 3M–3O). LacZ+ cells were observed in the interventricular septum and in the apical wall of the LV (Fig 3N). By E13.5 intramyocardial LacZ+ cells become apparent throughout the entire wall of the LV (Fig 4K–4N). At this stage, the anterior part of the RV still lacked LacZ expressing cells as opposed to the posterior part of the RV that now also contained a few LacZ + cells (compare Fig 4L with 4N). By E14.5, LacZ+ cells were found in the anterior and posterior region of the RV in accordance with distribution patterns of WT-1 (Fig 5F and 5G). LacZ expression was more pronounced in the LV than in RV, and more abundant in the posterior part of the RV than in the anterior part of the RV (Fig 5F–5I).

### Myocardial compaction shows regional differences between and within ventricles

**Fig 6:**
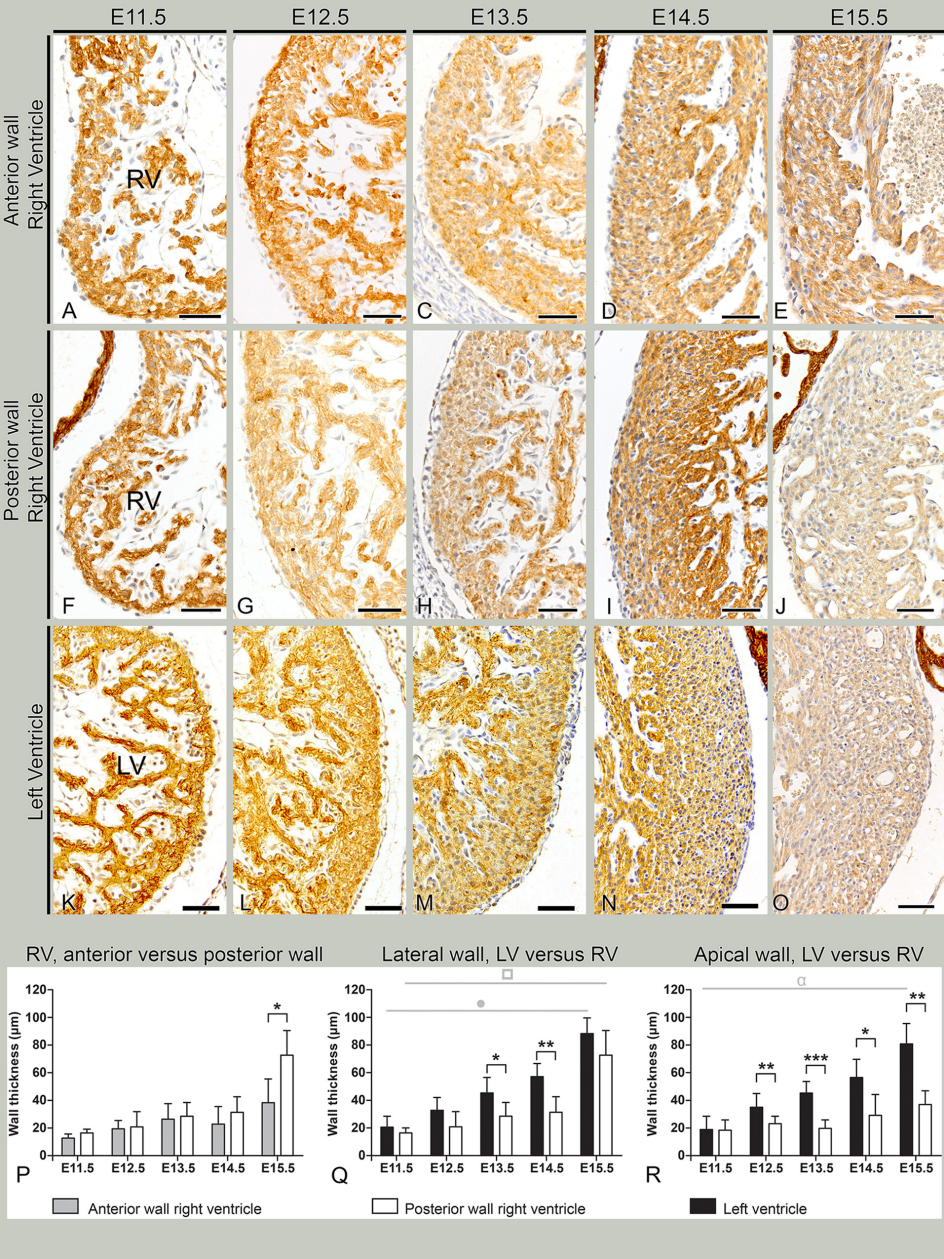
Ventricular compaction and wall thickness in sequential stages E11.5–15.5. A-E. Myocardial staining for myosin light chain 2a (MLC-2a) shows myocardial compaction and thickening of the anterior part of right ventricle (RV). F-J: Myocardial compaction and thickening of posterior part of RV. K-O: Myocardial compaction and thickening of the left ventricle (LV). The LV wall is thicker than RV, and the RV posterior wall appears thicker than anterior (compare sections E, J and O). P-R depict the myocardial wall thickness measurements of the RV and LV. Two distinction can be made in the measurements. One is the *anatomical region* of each ventricle: the anterior portion of the RV, the posterior portion of the RV or the posterior portion of the LV. The second distinction is the *location in the myocardial wall*: the lateral wall or the apical wall. P. The RV shows a significantly thicker posterior than anterior wall at E15.5. Q,R: RV and LV wall thickness. From E13.5, LV lateral wall (Q) is significantly thicker than RV, except for the latest stage (E15.5). For the apical wall (R) this difference is significant from E12.5. Grey line with circle (°) represents significant difference in LV thickness between E11.5-E13.5 and E14.5-E15.5, showing a gradual growth during development. Grey line with square (□) represents significant difference in RV thickness between 11.5–14.5 and E15.5, indicating a gradual ventricular growth during development. Grey line with alpha (α) represents significant difference in LV thickness between E11.5-E12.5 and E15.5. *p<0.05; **p<0.01; ***p<0.001. Bars: 50 μm.

A subpopulation of epicardial derivatives (expressing a.o. WT-1 and Tcf21) contributes to the interstitial fibroblast population of the heart [18], which in turn is a major contributing factor for proper ventricular growth [19]. Above, an asymmetric distribution of intramyocardial WT-1+ and Tcf21+ cells was demonstrated. As a next step we sought to determine ventricular compaction and wall thickness throughout development between ventricles in relation to these intramyocardial cell populations. Significant differences in ventricular compaction were observed between RV and LV per stage but also between regions (anterior versus posterior region, lateral versus apical wall) of the same ventricle throughout development. Normal myocardial compaction is summarized in Fig 6, for the anterior (Fig 6A–6E) and posterior part of the RV (Fig 6F–6J) and for the LV (Fig 6K–6O). At E11.5 both ventricles had a thin, uncompacted myocardial wall consisting of 1–2 layers of loosely organized cells, with the RV slightly smaller in size (Fig 6A, 6F and 6K). In subsequent developmental stages, ventricular compaction increased for both ventricles, however differences within and between ventricles were observed. The wall thickness of the LV increased significantly in both the lateral (Fig 6Q, black bars) and apical walls (Fig 6R, black bars).The RV showed a significant increase in wall thickness of the lateral wall compared to stage E15.5 (Fig 6Q, white bars) but not in the apical wall (Fig 6R, white bars). Within the RV, formation of a compact layer was more pronounced in the posterior part (Fig 6F–6J, white bars in Fig 6P) than in the anterior part (Fig 6A–6E, grey bars in Fig 6P) specifically at E15.5 (Fig 5P). No significant growth was observed within the anterior part of the RV throughout development (Fig 6P, grey bars). This is in accordance with more WT-1+ and Tcf21+ cells observed in the RV posterior wall compared to the anterior wall (Figs 4 and 5, quantified in Fig 2I). Comparing the left to the right ventricle at stage E11.5, there was no difference in wall thickness of both lateral and apical walls between the ventricles (Fig 6Q–6R, compare black (LV) with white (RV) bars). At E12.5 the LV became significantly thicker than the RV at the apical wall (Fig 6R **)** and from E13.5 also at the lateral wall (Fig 6Q). This data is in accordance with the quantitative analysis of intramyocardial WT-1 cells during development (Fig 2I). Based on these results, a relation between the amount of WT-1+ or Tcf21+ cells and myocardial compaction can be postulated.

### PDGFRα deficient embryos show less Tcf21 LacZ+ cells and diminished myocardial compaction

**Fig 7:**
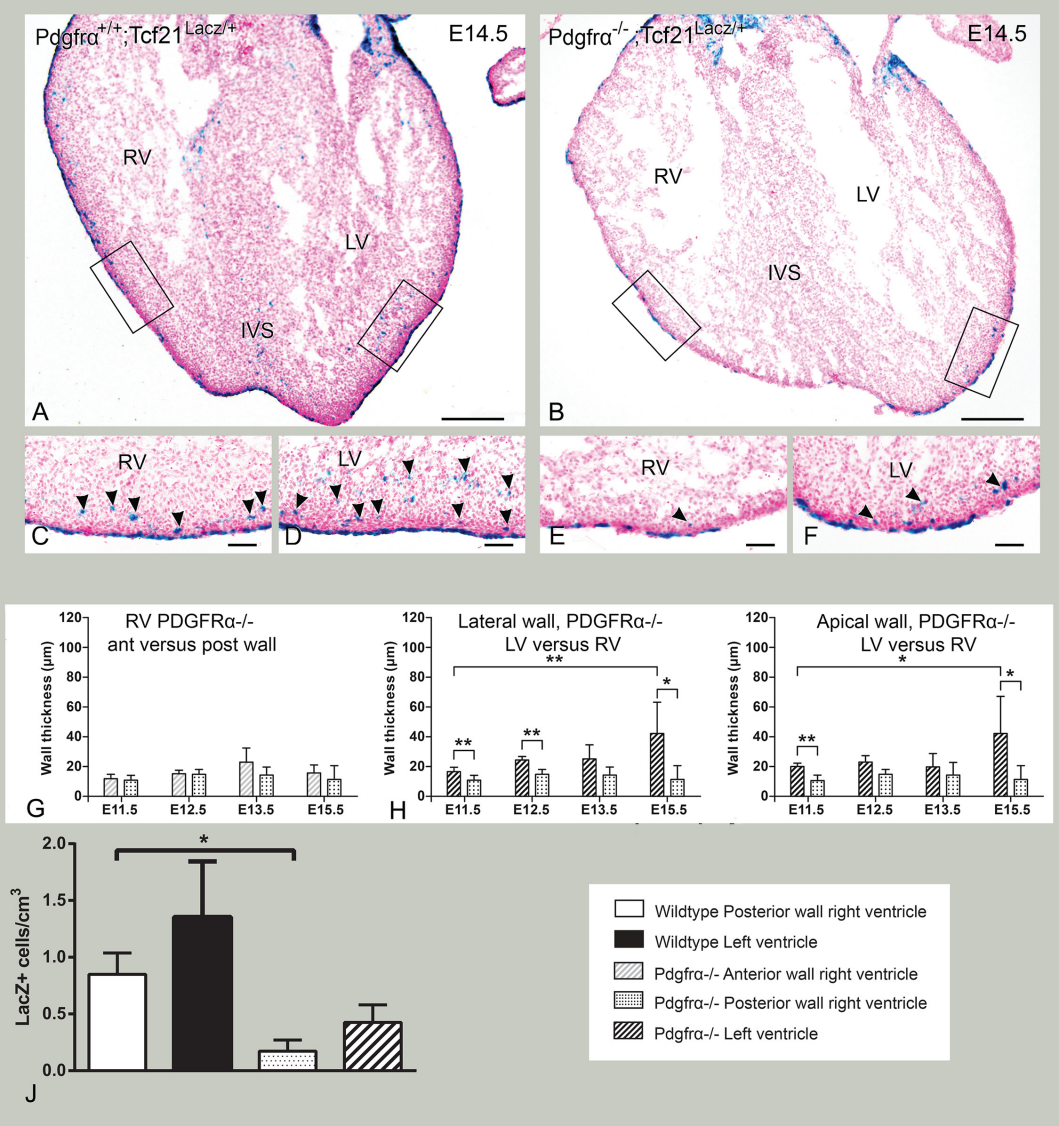
Myocardial compaction and Tcf21^lacZ/+^ distribution in PDGFRα knockout. A,B: PDGFR^+/+^;Tcf21^lacZ/+^ and PDGFRα^-/-^;Tcf21^lacZ/+^. C and D are enlargements of RV (C) and LV (D) in A; E and F enlargements of RV (E) and LV (F) in B. Epicardium formation is markedly disturbed and the number of lacZ+ cells severely reduced in PDGFRα^-/^; Tcf21^lacZ/+^. G-I: Quantification of myocardial thinning in PDGFRα knockout embryos. RV anterior and posterior wall (G) and LV and RV lateral wall (H) and apical wall (I). J: Quantification of LacZ+ population per cm^3^ in PDGFRα +/+ and-/- mice in E14.5 embryos. *p<0.05; **p<0.01; ***p<0.001. Bars: A-D: 200 μm, E-H:50 μm.

Embryos lacking PDGFRα have been associated with thin uncompacted myocardium[21] and PDGFRα signalling is essential for proper migration and differentiation of epicardial cells into fibroblast[21], however, there is no data on expression of Tcf21 in this model. PDGFRα^-/-^;Tcf21^LacZ/+^ embryos were studied to assess ventricular compaction and the distribution of the Lacz+ cells. These mice showed a disturbed epicardial covering of the ventricles, a severely thin compact myocardial layer as well as a diminished number of LacZ expressing cells (Fig 7A–7F). Ventricular compaction was severely affected in these mice (Fig 7G–7I). Specifically, a slight increase in LV compaction was observed at later stages than expected as seen in wildtype (compare Figs 6Q–6R to 7H–7I). For both the anterior and posterior portions of the RV, no increase in compaction was observed (Fig 7G–7I). Compared to wildtype, the LV and RV ventricular walls were significantly thinner both laterally and apically in the PDGFRα null embryos, confirming the thinning of the ventricular walls (S1A–S1C Fig, that contains a resume of data from Figs 6P–6R and 7G–7I, in order to show comparisons between WT and PDGFRα null embryos). As by E15.5 a slight increase in LV compaction was observed, the amount of lacZ+ cells PDGFRα^+/+^ and PDGFRα^-/-^ mice was determined at E14.5. The density of lacZ+ cells counted in the posterior region of the RV was significantly diminished in the PDGFRα^-/-^;Tcf21^LacZ/+^ mice compared to wildtype embryos (Fig 7J). Although an overall effect on both ventricles was observed, these results suggest that the RV is more severely affected by abnormal PDGFRα signalling compared to the LV.

In summary, regional differences in distribution of WT-1+ and Tcf21+ cells within the developing ventricles were observed in relation to the remodelling of the ventricular architecture shown by an increase in wall thickness.

## Discussion

In the adult heart differences in the LV versus RV are evident in gross morphology, myocardial compaction, function and pathology. Certain cardiomyopathies, including ventricular non-compaction and arrhythmogenic RV dysplasia tend to have a lateralized occurrence and treatment options for right and left heart failure are not uniform [29,30].

Isolated segments within the RV and LV can be affected in congenital heart disease, e.g.in pulmonary atresia with intact ventricular septum and hypoplastic left heart syndrome, where particularly the posterior inlet component [11,12] of the ventricles is insufficiently developed. To better understand the regional differences within the adult ventricles in relation to specific lateral pathologies, we evaluated differences in timing of epicardial sheet formation from the PEO and the cellular distributions from WT-1+ or Tcf21^LacZ/+^ cells to each ventricle separately and within different regions of the ventricles.

### Wilm’s tumor-1 (WT-1) expression and ventricular compaction

WT-1 is a transcription factor expressed in the PEO, epicardium and temporarily in EPDCs. Additionally, WT-1 expression has been observed in endothelial cells from E13.5 onwards [27]. Up to date, WT-1 expression has not been observed in smooth muscle cells within the developing ventricles [27]. In the current study, the distribution pattern of WT-1+ cells from E13.5 onwards is most probably a reflection of the fibroblast and endothelial cell population within the ventricular myocardium. WT-1 expression has previously been shown to co-localize with Tcf21 expression in approximately 70% of cells during embryonic heart development [31]. The cellular portion that does not show co-localization is likely to contribute to the endothelial cell population, which also expresses WT-1 [27]. Cardiac fibroblasts play an important role in ventricular compaction [32]. During development, epicardium interacts with the myocardium [16,24]. As the LV and RV have a different developmental background [8, 10], we postulate that this interaction differs between both ventricles. Epicardial covering was completed later in the RV, although the intramyocardial presence of WT-1+ cells in the RV lateral wall occurred somewhat earlier than in the LV with only a few WT-1+ cells in the apical wall. A previous chick-quail chimera study demonstrated that permissiveness for invading EPDCs is variable within the embryonic heart [33]. We surmise that the loose organization of RV myocardium may facilitate early invasion of EPDCs into the RV, but a molecular activated homing is also possible. From E13.5 onwards, the number of intramyocardial located WT-1+ cells increased dramatically, specifically within the LV. Previous studies demonstrated that invasion of EPDCs is essential for normal ventricular compaction [16,24]. EPDCs induce myocardial alignment and proliferation through cell-cell interaction [34] and are the primary source of cardiac interstitial fibroblasts, regulating myocardial proliferation [14,18]. In the current study, myocardial thickening started simultaneously with the presence of WT-1+ cells in the myocardium. To our knowledge, this is the first study to describe regional differences in ventricular distribution of WT-1+ cells, with more cells in the RV postero-lateral wall than in the anterior wall, and more in the LV than in de RV. We postulate that the lack of EPDCs would exert the strongest effects in the LV and RV posterior wall, although mechanistic studies are needed to confirm this.

### Regional differences in epicardial derivatives and ventricular disease

EMT is tightly regulated involving expression of several genes during specific time windows. Alteration of these genes results in severe cardiovascular malformations and embryonic lethality as in the WT-1 null mice [35]. PDGFRα-signalling is essential for formation of cardiac fibroblasts [17,28] and PDGFRα-knockout results in severe cardiac defects including an abnormally thin and spongy myocardium [21,36]. In our study, epicardial sheet formation was markedly disturbed and the number of Tcf21+ cells severely reduced in PDGFRα-knockout mice. A previous study already showed reduced amount of WT-1+ cells in PDGFRα null embryos and an overall higher expression of WT-1 protein, hypothesizing higher levels of expression per cell [21]. In the current study in the PDGFRα^-/-^;Tcf21^LacZ/+^ model, both RV and LV were affected, as both ventricular walls were thinner. Significantly less lacZ+ cells were present in the posterior part of the RV compared to the LV of the PDGFRα^-/-^;Tcf21^LacZ/+^.

In light of recent studies, it is becoming more apparent that the PEO, epicardium and EPDCs do not constitute a homogeneous cell population, but rather a complex of different subpopulations that contribute to specific sets of cells within the adult heart [17,31,37]. WT-1 has been linked to the regulation of epicardial EMT [38,39], while Tcf21 regulates the timing and differentiation of EPDCs into fibroblasts [31]. In the current study we also observed timing differences between the ventricles, as intramyocardial WT-1 cells were first observed in the RV while Tcf21^LacZ^ + cells were more apparent at a later stage in the LV. This timing discrepancy could in part explain the wall thickness differences observed, as Tcf21 has been previously linked to the myocardial fibroblast population [18,31], essential for myocardial proliferation and compaction. These differences can account for functional differences within the ventricles, indicating that not only the expression profile of the epicardium and its derivatives but also the location and timing of these populations is important in determining ventricular anatomy and function. Of interest, the RV posterior wall, corresponding to the inlet part of the RV, is affected in congenital heart diseases such as pulmonary atresia with intact ventricular septum [12]. Although mechanistic studies in disease models are mandatory, it is tempting to speculate that a preference of ventricular disease to occur in specific regions might relate to the observed regionalized findings of WT-1+ and Tcf21+ cells in the ventricles.

### Functional relevance of EPDCs for the treatment of cardiac disease

RV dysfunction is recognized in adult patients with congenital or acquired heart disease [2]. Most therapies have limited or even adverse effects on RV function. For example, in patients with transposition of the great arteries and a systemic RV, treatment with angiotensin receptor blockers does not improve ventricular function, contrary to patients with LV disease [3,4,40].

Pluripotent EPDCs may have potential for cell-based therapies [41–44] when the developmental paracrine function of EPDCs can be reactivated in adult life [45,46]. As our evaluation indicates that epicardial influence induces different patterns in RV and LV, future studies need to explore the mechanisms that guide EPDC-myocardium interaction taking right-left and regional differences into account.

## Conclusion

The RV is covered later by epicardium and less densely than the LV. After migration, right-left differences are also noted. WT-1+ cells are present slightly earlier in the RV postero-lateral wall than in the LV lateral wall. During further development, WT-1+ and Tcf21+ cells will be more abundant in the LV myocardium, and more in posterior than in anterior regions of the RV. Compact myocardial layer formation runs parallel with the presence of WT-1+ and Tcf21+cells and is more pronounced in the LV than RV, and in the RV posterior than in the anterior wall. In PDGFRα^-/-^;Tcf21^LacZ^ mouse embryos, covering of epicardium and the amount of LacZ+ cells is severely reduced, especially in the RV, resulting in thin, uncompacted ventricles. These results may provide a developmental explanation for the more prominent hypoplasia of the posteriorly located inlet compartment of the ventricles in specific kinds of congenital heart disease and for the lateralized occurrence of cardiomyopathies. Future research to elucidate the molecular mechanisms involved in EPDC-myocardium interaction in left versus right ventricles, specifically in mutant models with lateralised (congenital) heart disease phenotypes, is essential as a next step to resolve the etiology and form a basis for a cell based approach to these specific forms of cardiac disease.

## Materials and Methods

### Mice Lines

Handling of all animals was according to the Guide for Care and Use of Laboratory Animals, as published by the NIH and approved by the relevant local animal ethics committee and approved by the animals experiment committee of the Leiden University Medical Center (Permit Numbers 11211 and 10177).

Wild type mouse embryos were obtained from the CLB-Swiss strain. Wt1^CreERT2/+^mice and the Cre-activated reporter Rosa26^fsLz/+^ mice were studied [47]. Tamoxifen was injected at Embryonic day (E)10.5 into pregnant Wt1CreERT2/+; *Rosa26fsLz/+* mouse to induce Cre expression. X-gal stainings were performed at E12.5 and E13.5 (N = 5 per stage) to study the migration into the myocardium of the WT-1 lineage cell population. Mice were kindly provided by Dr. William Pu and Dr. Bin Zhou (Harvard University).

To investigate the effect of abnormal EPDC formation and function, a PDGFRα-null mouse and Tcf21^lacZ/+^ reporter mice were studied kindly provided by Dr. Steven Bleyl (University of Utah) and Dr. Michelle Tallquist (University of Hawaii) respectively [36,48]. Previous studies have shown that some of these mutants can present with severe growth retardation [21]. Littermates that deviated more than E0.5 from their estimated embryonic stage were excluded from the study. β-Galacotosidase stainings were performed in both PDGFRα^+/+^;Tcf21^lacZ/+^ and PDGFRα^-/-^; Tcf21LacZ/+ [18].

E9.5 up tp E15.5 (N = 3 per stage) embryos were processed for (immuno)histochemical and morphometric analyses. Handling of all animals was according to the Guide for Care and Use of Laboratory Animals, as published by the NIH and approved by the Committee of Animal Welfare of the Leiden University Medical Center. Pregnant mice were euthanized by cervical dislocation. The day the vaginal plug was detected, was designated embryonic day (E)0.5. After fixation in 4% paraformaldehyde in phosphate buffered saline(0.1 M, pH 7.2) and dehydration embryos were embedded in paraffin, sectioned transversely(5μm) and serially mounted on glass slides.

### Immunohistochemical procedures

Immunohistochemical staining was performed as decribed previously[20–22,49] for primary antibodies against Myosin light chain-2a (MLC-2a, 1/6000, gift from S.W. Kubalak, Charleston, SC); cardiac Troponin I (cTnI, 1/800, Santa Cruz Biotechnology, CA, SC-15368) and Wilm’s tumor-1 (WT-1, 1/1000, Santa Cruz Biotechnology, sc-192). Slides were incubated with biotin-conjugated secondary antibody goat-anti-rabbit-biotin(1/200; Vector Laboratories, USA, BA-1000.The signal was amplified using ABC-reagent(Vector Laboratories, PK 6100), visualized with 3–3’di-aminobenzidine tetrahydrochloride(DAB, Sigma-Aldrich, D5637), and counterstained with 0.1% hematoxylin(Merck, Darmstadt, Germany). β-gal stainings were performed as previously described[49]. After overnight incubation at 3700B0C with 5-bromo-4chloro-3-indolyl-β-D-galactopyranoside (X-gal; 1mg/ml), hearts were stained against β-galactosidase (MP Biochemicals).

For immunofluorescence stainings, sections were incubated with primary antibodies against WT-1 (Wt1, clone 6F-H2, Millipore, CN-05-753 and 1/500 Abcam, ab899901) and cTnI (HyTest Ltd, CN-4T21_2) overnight at 4°C. Tyramide Signal Amplification (PerkinElmer, CN- NEL749A001KT) was used to amplify the signal of the primary antibody against WT-1. Slides were incubated with Alexa-conjugated secondary antibodies appropriate to the specific species: for WT-1 Alexa Fluor 488 streptavidin (Invitrogen, CN-S-32354) and for cTnI Alexa Fluor 555 (Invitrogen, A-21432). All sections were mounted with ProLong Gold antifade reagent (Invitrogen, CN-P36930) with DAPI.

### Three-dimensional reconstruction

To address the timing of epicardial covering of the ventricles, expression of WT-1 from E9.5, E9.75, E10.5, E10.5 and 11.5 was 3D visualized. Micrographs(10X) of serial sections were processed using AMIRA as described previously [22] (Template Graphics Software, San Diego, CA). The myocardium, the arterial and the venous pole were reconstructed in MLC-2a or cTnI stained sections, after which WT-1 expressing cells were superimposed. The nucleus of each WT-1+ cell was separately indicated. A subset of reconstructions were converted to 3D interactive PDFs, available online. Different functions are available in the pdf file (e.g. buttons to show or hide each structure) and have been modified after [50].

### Quantitative measurements

Quantification of the number of WT-1+ cells in the epicardial, subepicardial and intramyocardial layer was obtained by analysis of WT-1 stained heart sections of hearts from E12.5 to E15.5 (n = 3 to 5 per group). In each heart, a posterior section was selected that included the LV and RV, the interventricular septum and the atrioventricular transition. As the RV consists of an anterior and posterior compartment [51], separate anterior photographs (proximal region to the OFT) were taken, but not for the LV, as the aortic valve is in continuity with the mitral valve, complicating an antero-posterior distinction. Three non-serial sections separated by at least 25μm were quantified per region of interest. Regions from the LV, the posterior compartment of the RV and the anterior compartment of the RV were analysed. Quantification was achieved using the ImageJ software (http://imagej.nih.gov/ij/, 1997–2011). A detection threshold was selected and the amount of WT-1+ nuclei was quantified per section. The absolute quantification was corrected for the measured volume in each region. The same quantitative method was used on the same sections were the epicardial and subepicardial region was removed to obtain the quantification of the intramyocardial WT-1+ fraction. The epicardial and subepicardial fraction was obtained by subtracting the intramyocardial fraction from the total fraction.

Wall thickness was assessed in the LV and RV apical and lateral walls. The RV was separated in an anterior and in a posterior region. Micrographs were made of 3–6 different wildtype (E11.5–15.5). Wall thickness was evaluated in each section by 2 measurements: one triplicate at the lateral wall, and one triplicate apically. Analyses were performed by two independent and blinded observers (MRMJ, RVS) using ImageJ(http://imagej.nih.gov/ij/, 1997–2011). Quantification of the fibroblast population in PDGFRα^-/-^;Tcf21^LacZ/+^ and PDGFRα^+/+^;Tcf21^LacZ/+^ was performed by counting the amount of Tcf21-derived Lacz-expressing cells within the ventricular myocardium in E14.5 embryos. Data was normalized to the total ventricular volume and is presented as number of cells per cm^3^.

### Statistical analysis

Analysis was conducted using GraphPad Prism 6.00 for Windows software (GraphPad Software, La Jolla, California). Comparison between groups was accomplished using unpaired Student’s t-test when the data showed a normal distribution or using the Mann-Whitney U test when this was not the case. Data are represented as mean±SD.

## Supporting Information

S1 File 3D pdf file. Epicardial covering at E9.75, E10 and E10.5. Interactive 3D file showing epicardial covering at 3 early developmental stages (E9.75, E10 and E10.5). A user manual is provided after opening the pdf file.
(PDF)

S1 Fig Myocardial compaction: comparison between wildtype and PDGFRα knockout. A-C: Quantification of myocardial thinning. RV anterior and posterior wall (A) and LV and RV lateral wall (B) and apical wall (C) in wildtype and complete PDGFRα knockout embryos. Wildtype graphs from Fig 6P–6R have been merged with PDGFRα knockout graphs from Fig 7G–7I to better depict the differences. *p<0.05; **p<0.01; ***p<0.001.
(TIF)
