# Supplementary material for: Regional differences in WT-1 and Tcf21 expression during ventricular development: implications for myocardial compaction
Source: PLoS One. 2015 Sep 21;10(9):e0136025. doi: 10.1371/journal.pone.0136025 (PMC4577115; doi:10.1371/journal.pone.0136025)
Supplement: S1 File — Epicardial covering at E9.75, E10 and E10.5. Interactive 3D file showing epicardial covering at 3 early developmental stages (E9.75, E10 and E10.5). A user manual is provided after opening the pdf file. (PDF) [file pone.0136025.s001.pdf]

Instructions for online interactive 3D PDF Supplemental Figure 1 (9.75-E10.5)

Requiered settings:
To be able to correctly use this interactive PDF file, please use the settings provided which have been adapted and slightly modified after De Boer *et al*[1].

- Use Adobe Acrobat® 9.3 or higher
- Follow the menu tree: Edit → Preferences→ 3D & Multimedia → 3D Tool Options and change the following settings:
    - For “Open Model Tree on 3D activation” choose “No”
    - For “Default Toolbar State” choose “Hidden”
    - Disable “Show 3D Orientation Axis”
- Follow the menu tree: Edit → Preferences → 3D & Multimedia → Auto-Degrade Options:
    - ‘Optimalization Scheme for Low Framerate” choose “None”
- Follow the menu tree: Edit → Preferences→ Javascript
    - Make sure “Enable Acrobat Javascript” is switched on

How to use this 3D PDF
Activate: click on the reconstruction
Move: hold the left mouse button and move the mouse
Zoom: scroll up and down with the mouse wheel or hold the right mouse button and move up and down with the mouse
Pre-programmed views: on the right side of the reconstruction under “Views”, four pre-programmed views are available, which are activated by clicking on the small thumbnail of the view
Show, hide or make structures transparent: The different structures (e.g. the Epicardium) that make up the reconstruction can be shown, hidden or made transparent by clicking on the “+” (=show), “+/-” (=transparent) or “-” (=hide) buttons underneath the desired structure (see below).

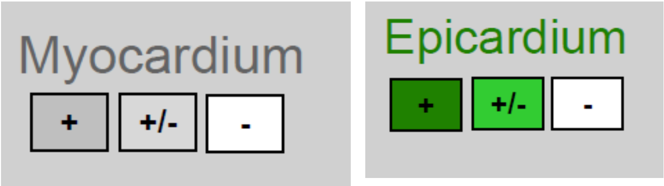

Move between the 3 different reconstructions: Three different developmental stages have been reconstructed. To move between the different stages, buttons have been provided. They can be found at the top left corner. Press the button of the desired stage to view that reconstruction (see below). You can also scroll up or down from page to page.

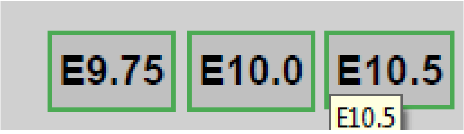

Description of the reconstructions
In this pdf, three mouse embryonic hearts have been reconstructed from stages E9.75, E10.0 and E10.5. The proepicardial organ (PEO) and epicardial cell population can be studied during these developmental stages.

[1] De Boer BA, Soufan AT, Hagoort J, Mohun TJ, van den Hoff MJB, Hasman A, Voorbraak FPJM, Moorman AFM, Ruijter JM. 2011. The interactive presentation of 3D information obtained from reconstructed datasets and 3D placement of single histological sections with the 3D portable document format. Development 138:159–167.

Mouse E9.75

E9.75 E10.0 E10.5

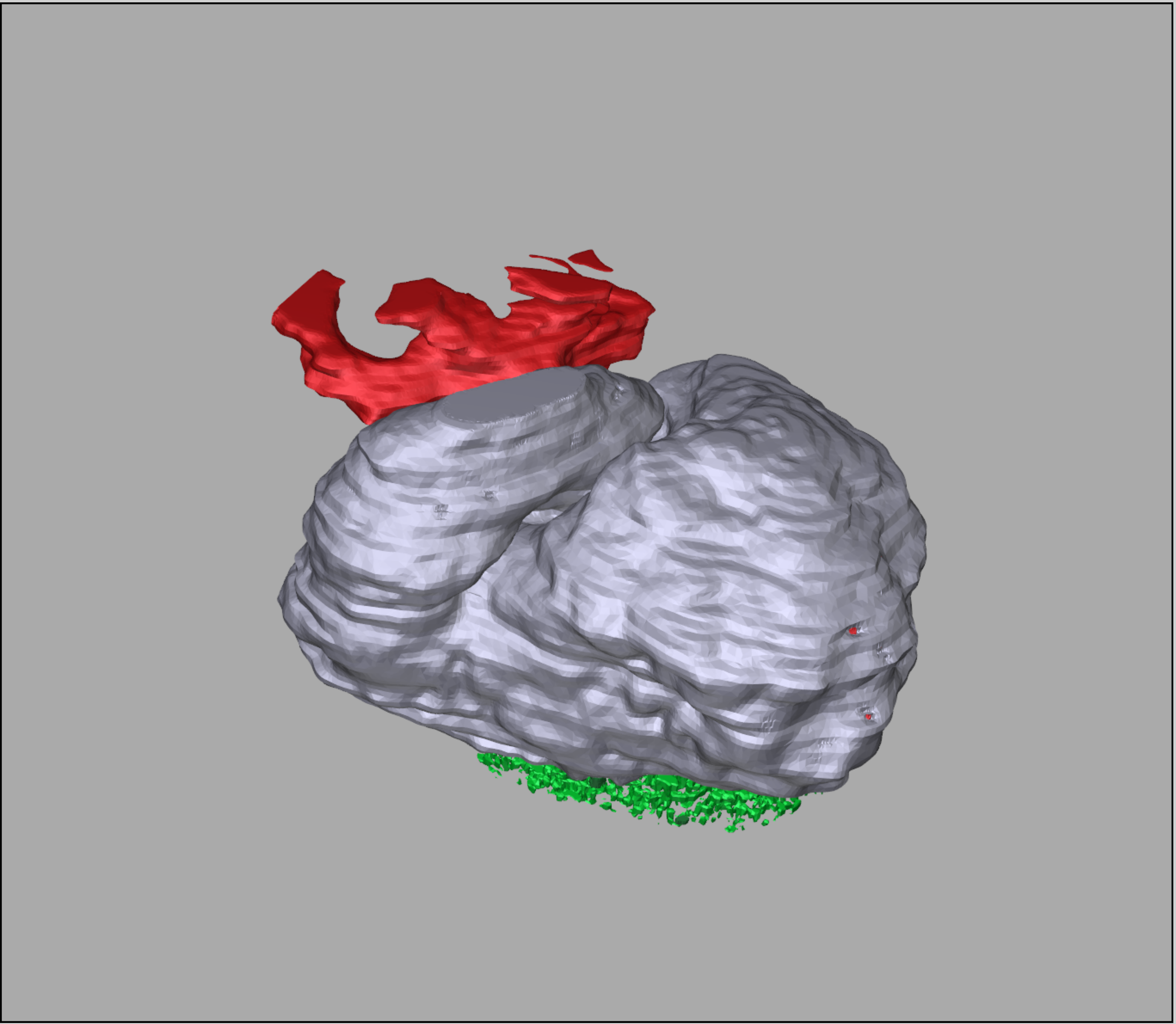

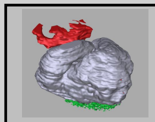
Anterior view

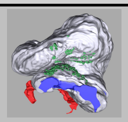
Posterior view

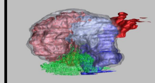
L-lateral view

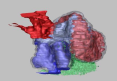
R-lateral view

Myocardium

+ +/- -

Cushion

+ +/- -

Lumen outflow

+ +/- -

Lumen inflow

+ +/- -

PEO

+ +/- -

Epicardium

+ +/- -

Mouse E10.0

E9.75 E10.0 E10.5

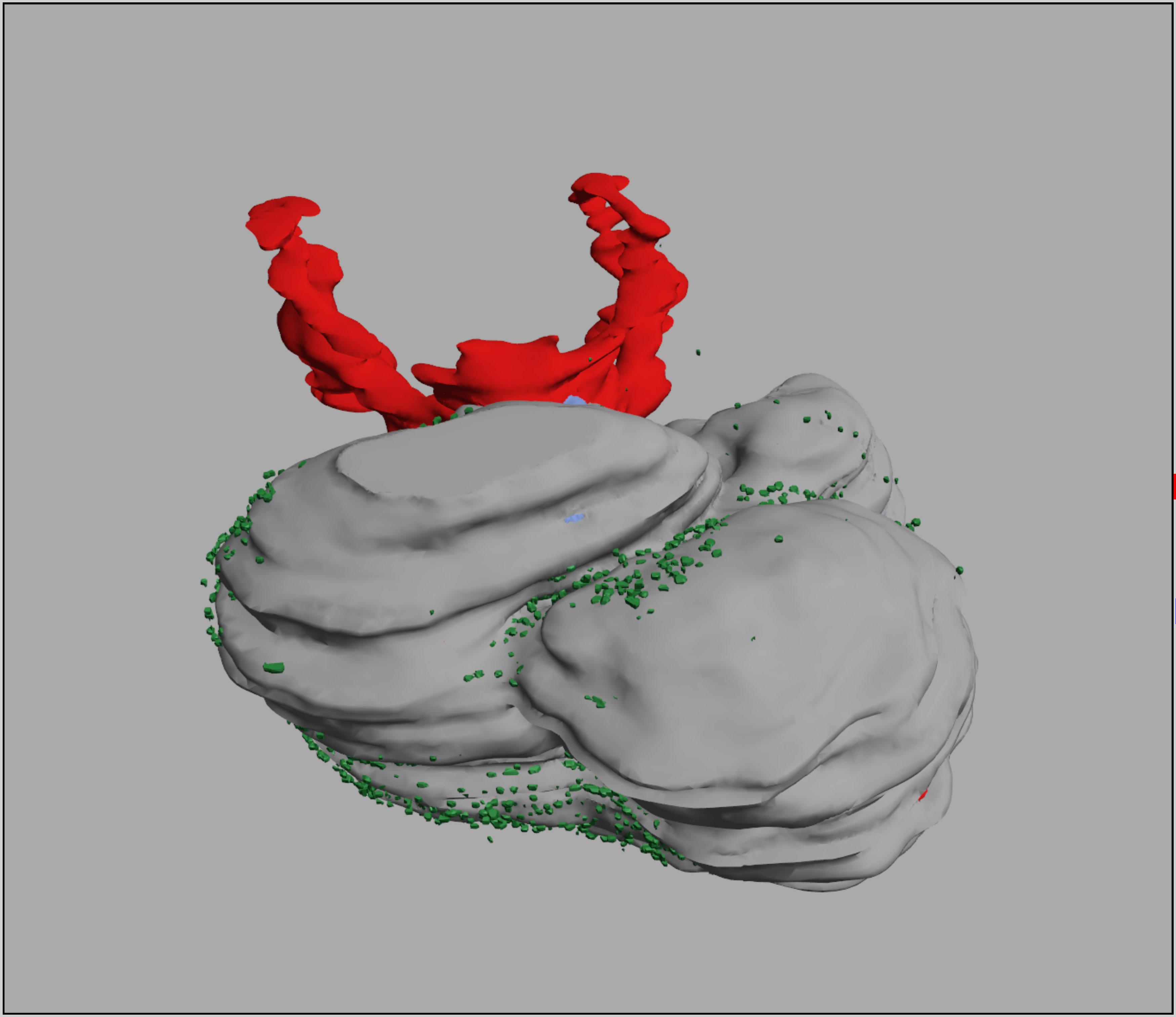

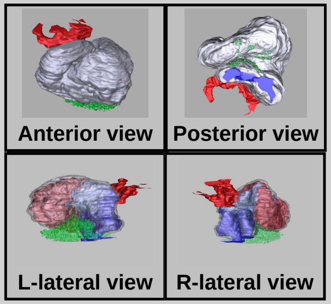

Myocardium

+ +/- -

Cushion

+ +/- -

Lumen outflow

+ +/- -

Lumen inflow

+ +/- -

PEO

+ +/- -

Epicardium

+ +/- -

Mouse E10.5

E9.75 | E10.0 | E10.5

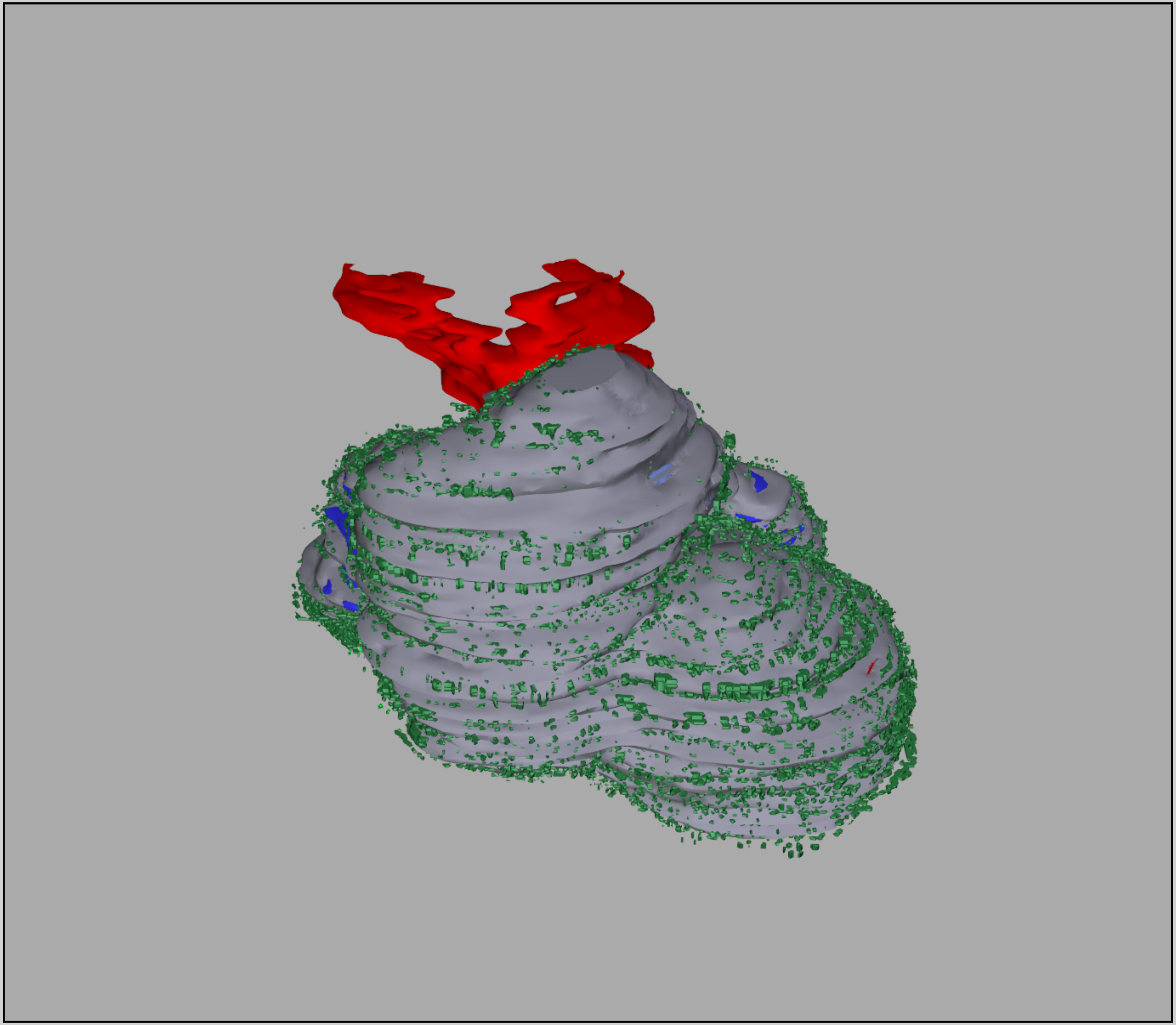

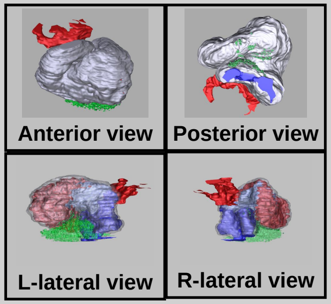

Myocardium

+ | +/- | -

Cushion

+ | +/- | -

Lumen outflow

+ | +/- | -

Lumen inflow

+ | +/- | -

PEO

+ | +/- | -

Epicardium

+ | +/- | -
