# Supplementary figures and images for: Regional differences in WT-1 and Tcf21 expression during ventricular development: implications for myocardial compaction
Source: PLoS One. 2015 Sep 21;10(9):e0136025. doi: 10.1371/journal.pone.0136025 (PMC4577115; doi:10.1371/journal.pone.0136025)

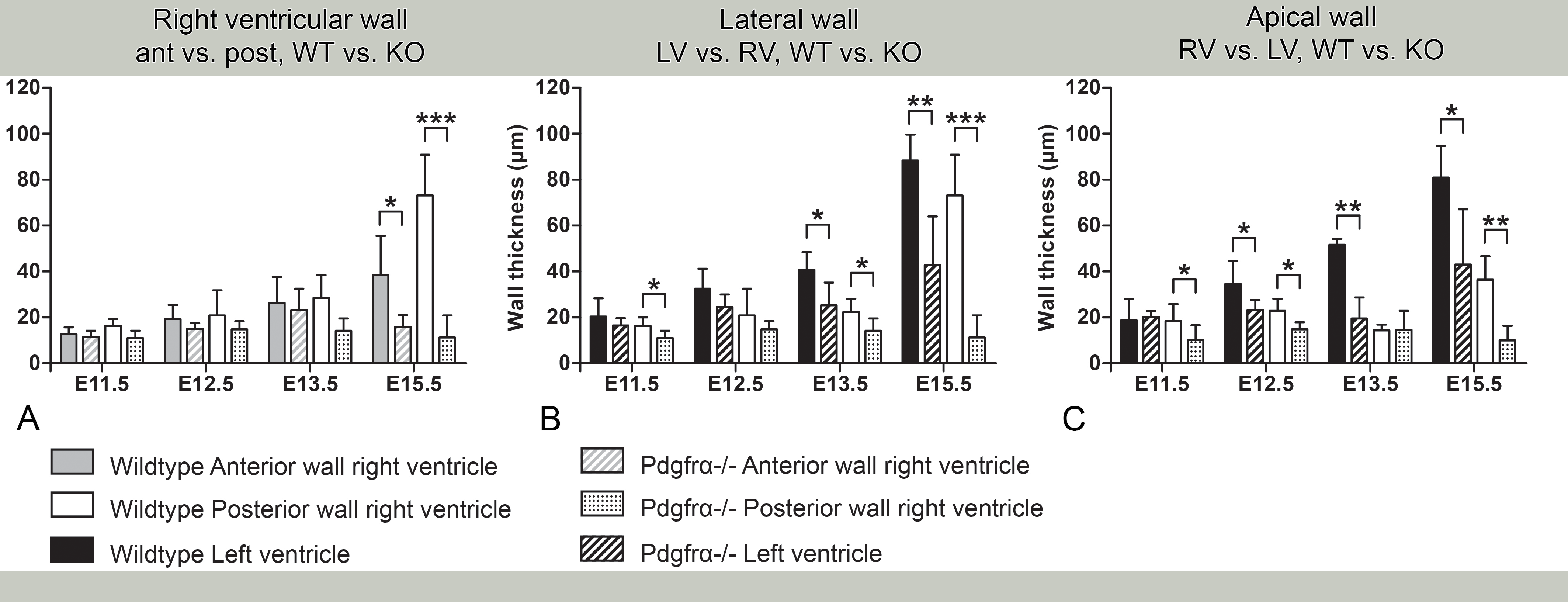

Supplement: S1 Fig — A-C: Quantification of myocardial thinning. RV anterior and posterior wall (A) and LV and RV lateral wall (B) and apical wall (C) in wildtype and complete PDGFRα knockout embryos. Wildtype graphs from Fig 6P–6R have been merged with PDGFRα knockout graphs from Fig 7G–7I to better depict the differences. *p<0.05; **p<0.01; ***p<0.001. (TIF) [file pone.0136025.s002.tif]
